# Patient Adherence to Follow-Up Recommendations Following Cryotherapy for Treatment of High-Grade Cervical Dysplasia

**DOI:** 10.7759/cureus.30154

**Published:** 2022-10-10

**Authors:** Taylor Lendrum, Meredith Alston, Elaine Stickrath, Karilynn Rockhill

**Affiliations:** 1 Obstetrics and Gynecology, University of Colorado, Aurora, USA; 2 Obstetrics and Gynecology, Denver Health Medical Center, Denver, USA; 3 Obstetrics and Gynecology, University of Colorado, Steamboat Springs, USA; 4 Epidemiology and Public Health, Denver Health Medical Center, Denver, USA

**Keywords:** cervical dysplasia, hpv, follow-up, cryotherapy, dysplasia

## Abstract

Objective

Determine the rate of patient adherence to follow-up recommendations after cryotherapy for high-grade cervical lesions, and identify patient characteristics associated with adherence to follow-up.

Methods

This is a retrospective case series from May 2016 to June 2018 of patients who underwent cryotherapy for high-grade dysplasia at a single academic safety-net hospital. Patient demographics and clinical information were abstracted from the electronic medical record. All patients were recommended to follow up with Pap and high-risk human papillomavirus (HPV) testing 12 months after their procedure. The primary outcome was patient adherence to these recommendations. Descriptive statistics and statistical testing were utilized to compare adherence by demographic and clinical characteristics. A multivariable logistic model was used to preliminarily look at potential factors associated with increased odds of adherence. We further described the proportion of follow-up testing among those patients who adhered to recommendations.

Results

One hundred and forty-three patients met the inclusion criteria. The adherence percentage was 60.1% (95% CI: 51.6, 68.2). Only employment was associated with follow-up among demographic variables reviewed (p=0.039). Of those who were adherent with follow-up, 4.7% (4/86) had high-grade findings on follow-up Pap testing, and 56.9% (49/86) had negative cytology and negative HPV testing.

Conclusion

Adherence to follow-up recommendations for the following cryotherapy for high-grade dysplasia within our system was poor, and demographic factors were generally not associated with adherence to follow-up. Given these findings, cryotherapy should be used with caution.

## Introduction

Treatment for high-grade cervical dysplasia, defined as cervical intraepithelial neoplasia (CIN) 2 or greater, may be recommended to prevent progression to cervical cancer [1-3]. Options include excisional procedures as well as ablative techniques. Excisional procedures typically include loop electro excisional procedures (LEEP) and cold knife conization (CKC), while ablative techniques include laser and cryotherapy. These methods effectively treat high-grade cervical dysplasia with no significant difference in efficacy based on a systematic review [4-6].

These therapies have advantages and disadvantages, which may guide providers and patients towards one treatment over the other. There is inconclusive data regarding excisional procedures' impact on preterm delivery risk [7]. Some studies suggest an approximately a 2-fold increased risk in subsequent preterm delivery. This risk increases with the size and number of excisions [8-10]. Other studies posit that this risk is due to dysplasia or HPV alone [11]. Given the potential increased risk of preterm delivery with an excisional procedure, ablative therapy has been considered an alternative treatment option after counseling in reproductive-age women [12].

Cryotherapy is also used in international low-resource settings [3]. In addition to changing guidelines regarding the most appropriate therapy, adherence to follow-up in patients treated with cryotherapy has not been thoroughly evaluated. Most studies aim to compare the persistence of dysplasia or high-risk human papillomavirus (HPV) after treatment (Pubmed Search/"Cryotherapy" AND "Dysplasia"/ 232 results/ 3/29/21). This is particularly relevant given that ASCCP's most recent risk-based management guidelines continue to include ablative therapy as an acceptable treatment option for high-grade cervical dysplasia [13]. Follow-up surveillance is vital, as with any therapy for cervical dysplasia and HPV. 

Our study assesses patient adherence to follow-up recommendations after cryotherapy for high-grade cervical lesions at Denver Health Medical Center (DHMC), an academic urban safety net institution.

This abstract was presented at ACOG Annual Clinical and Scientific Meeting 2021.

## Materials and methods

This is a retrospective case series of patients who underwent cryotherapy for high-grade dysplasia at DHMC between May 2016 and June 2018. Patients were identified through the electronic medical record (EMR). Patients were candidates for cryotherapy if they had a diagnosis of CIN2+, a negative endocervical sample, an adequate colposcopy, and had not yet completed childbearing per institutional policy. Patients with human immunodeficiency virus (HIV), lupus, or who became pregnant during the follow-up interval were excluded. Patients were selected for cryotherapy based on provider evaluation and discussion of treatment options with the patient.

Demographic variables were extracted from the EMR. Race/ethnicity information was obtained from the EMR report function. Of note, race/ethnicity obtained in this fashion is subject to bias, given that this data can be inconsistently uploaded. A chart review was conducted, and follow-up Pap and HPV testing results were extracted. The ASCCP recommended follow-up interval after ablative therapy during the study period was 12 months [MASSAD]. We defined adherence as completion of Pap and HPV testing within 10-18 months of the cryotherapy procedure based on the ASCCP guideline. Colorado Multiple Institutional Review Board deemed this protocol 20-0202 exempt for review.

Two full-time registered nurses are responsible for all cervical screening follow-up and care coordination in the DHMC system. Patients are contacted up to three times via phone and letter, with up to four weeks in between, to schedule a visit. The patient is considered lost to follow-up only if these attempts at contact were completed.

The binomial proportion and 95% confidence interval (CI) for adherence were determined along with the mean follow-up time. Descriptive statistics of patient and clinical characteristics by adherence were calculated; comparisons were conducted through Fisher's Exact Tests and Satterthwaite or Wilcoxon two-sample tests based on data distribution. Given case series studies do not have a pre-specified hypothesis, a multivariable logistic model was used to preliminarily look at potential factors associated with increased odds of adherence. Based on 143 patients available for modeling, we chose variables for the multivariate logistic regression to include a mix of patient and clinical characteristics that were not correlated. Adjusted odds ratios (OR) for the odds of not adhering compared to adherence to follow-up are presented for 5-year increments of age, race/ethnicity, employment status, and contraception use, as well as the global test for the significance of each factor in the model. For modeling, unknown or missing categories were collapsed for more stable estimates, and contraception use was defined as any contraception method. Finally, the follow-up Pap and HPV testing results among patients who completed follow-up within the recommended 10-18 months of cryotherapy were described.

## Results

**Figure 1:**
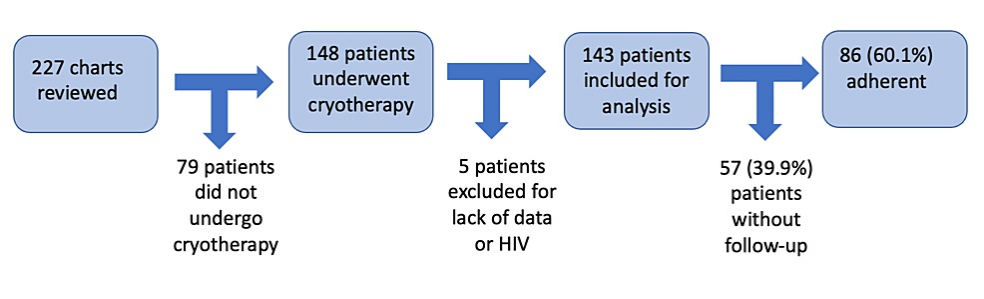
Patients included for analysis and adherence rate.

One hundred forty-three patients were included in the analysis after completing a chart review of 227 patients, as demonstrated in Figure 1. Follow-up data between 10 to 18 months was available for 86 patients, demonstrating an adherence percentage of 60.1% (95% CI: 51.6, 68.2), as shown in Figure 1. Among those who adhered to follow-up (N=86): the mean follow-up time was 13.1 months (standard deviation =1.8). 57 (39.9%, 95% CI: 31.8, 48.4) patients did not adhere to follow-up within the Denver Health System. The mean age of patients was 29 years old. The demographic makeup included 47.6% White and 35.7% Hispanic patients, as reported in the EMR. The majority of patients spoke English (79.7%) and did not smoke (61.3%) (Table 1).

**Table 1:** Comparing patient demographics & clinical characteristics among patients who adhered to recommendations to those who did not. ¬ LARC, long active reversible contraception; DMPA, depot-medroxyprogesterone acetate; HPV, Human papillomavirus; EMR, electronic medical record. Data were N (%) unless otherwise specified. a: Other is not a convenience group but what was reported from the EMR. b: Unknown, as it relates to race/ethnicity, indicates this data was not completed. * indicates significance.

Demographics	Total Cohort N=143	Adherence to Follow-up N=86	No Adherence to Follow-up N=57	p-value
Age (years)				NS
Mean (SD)	29.1 (4.2)	28.9 (4.1)	29.4 (4.4)	
Gravidity				NS
Median (IQR)	2 (1, 3)	2 (1, 3)	2 (1, 3)	
Missing	14 (10.9)	8 (10.3)	6 (11.8)	
Parity				NS
Median (IQR)	1 (0, 2)	1 (0, 2)	1 (0, 3)	
Missing	14 (10.9)	8 (10.3)	6 (11.8)	
Race/Ethnicity				NS
Hispanic	51 (35.7)	34 (39.5)	17 (29.8)	
Other^a^	3 (2.1)	1 (1.2)	2 (3.5)	
Unknown^b^	21 (14.7)	9 (10.5)	12 (21.1)	
White	68 (47.6)	42 (48.8)	26 (45.6)	
Language				NS
English	114 (79.7)	66 (76.7)	48 (84.2)	
Spanish	23 (16.1)	17 (19.8)	6 (10.5)	
Birthplace				NS
United States	99 (73.3)	60 (73.2)	39 (73.6)	
Mexico	21 (15.6)	15 (18.3)	6 (11.3)	
Other	15 (11.1)	7 (8.5)	8 (15.1)	
Employed				*
Yes	82 (57.3)	54 (62.8)	28 (49.1)	
No	9 (6.3)	2 (2.3)	7 (12.3)	
­	52 (36.4)	30 (34.9)	22 (38.6)	
Smoking Status				NS
Current	23 (16.2)	14 (16.3)	9 (16.1)	
Former	32 (22.5)	16 (18.6)	16 (28.6)	
Never	87 (61.3)	56 (65.1)	31 (55.4)	
Clinical Characteristic	Total Cohort N=143	Adherence to Follow-up N=86	No Adherence to Follow-up N=57	p-value
Contraception Type				NS
LARC	65 (45.5)	41 (47.7)	24 (42.1)	
Condom	10 (7.0)	5 (5.8)	5 (8.8)	
Surgically Sterile	8 (5.6)	4 (4.7)	4 (7.0)	
Combined Hormonal	21 (14.7)	11 (12.8)	10 (17.5)	
DMPA	7 (4.9)	6 (7.0)	1 (1.8)	
None	32 (22.4)	19 (22.1)	13 (22.8)	
HPV Vaccine Series				NS
Yes	27 (18.9)	20 (23.3)	7 (12.3)	
No	116 (81.1)	66 (76.7)	50 (87.7)	
History of Dysplasia Treatment				NS
Ablative	1 (0.7)	0 (0.0)	1 (1.8)	
Excision	3 (2.1)	3 (3.5)	0 (0.0)	
None	138 (97.2)	83 (96.5)	55 (98.2)	

**Figure 2:**
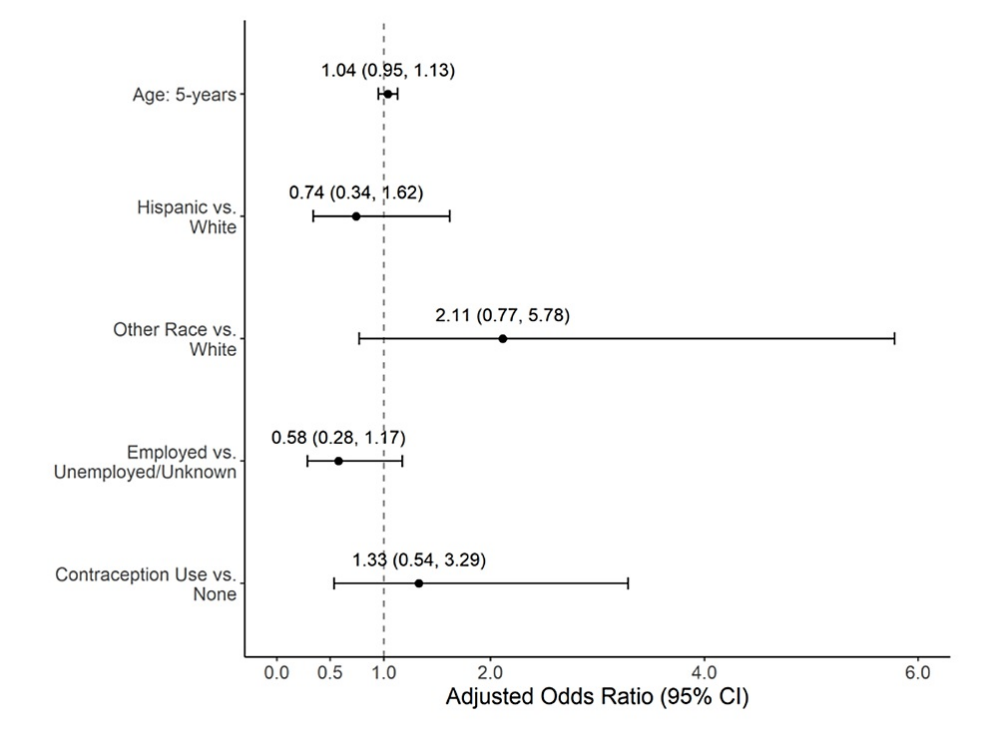
Odds ratios for the multivariate logistic regression model comparing demographics and clinical characteristics for patients who adhered to follow-up compared to no adherence.

Analyses comparing characteristics of patients by adherence demonstrated that those who completed follow-up were not different across many patient characteristics (p>0.05 for all) except for employment. Those who adhered were more likely to be employed (p=0.039, Table 1). There were no differences in adherence in the clinical characteristics (Table 1). In the logistic regression model, none of the factors were associated with a lack of adherence. Adjusted odds ratios for each factor are shown in Figure 2.

For patients that were adherent, on follow-up testing, the majority of patients had normal cytology and negative HPV (N=49; 57.0%), 4.7% (4/86) had high-grade cytology, and 4.7% had persistent HPV (4/86) (Table 2).

**Table 2:** Cytology and pap results for those patients who adhered to follow-up recommendations after cryotherapy. NILM, Negative for Intraepithelial Lesion; ASCUS, Atypical Squamous Cells of Undetermined Significance; LSIL, Low-grade Squamous Intraepithelial Lesion; ASC-H, Atypical Squamous Cells, Cannot Rule Out High-Grade Squamous Intra-epithelial Lesion; HSIL; High-grade Squamous Intraepithelial Lesion.

Result (Cytology/HPV)	Adherence to Follow-up N=86 N (%)
NILM/Neg	49 (57.0)
ASCUS/Neg	12 (14.0)
ASCUS/Pos	1 (1.2)
LSIL/Neg	14 (16.3)
LSIL/Pos	2 (2.3)
ASC-H/Neg	2 (2.3)
ASC-H/Pos	1 (1.2)
HSIL/Neg	1 (1.2)
Missing	4 (4.7)

## Discussion

The overall adherence rate to follow-up recommendations after cryotherapy within our system was 60.1%, raising concern. The advantage of cryotherapy is that it is less invasive than an excisional procedure; however, there is no pathologic specimen. Although systematic review demonstrates no difference, some studies suggest that short-term reoccurrence is increased with cryotherapy compared to excisional procedures [14]. Follow-up surveillance is critical for patients with cervical dysplasia after undergoing treatment, and we would argue that follow-up is even more critical for patients having undergone cryotherapy. Given the poor follow-up rate after this ablative procedure at DHMC, it brings into question if this is an acceptable treatment alternative in patients with pre-cancerous lesions of the cervix. 

Previous studies have evaluated the follow-up adherence to treatment and surveillance of those patients diagnosed with CIN 2+ [15-17]. Chase et al. described 45% adherence after diagnosis of CIN 2 or 3 during colposcopy [16], whereas Alston et al. described 89% adherence after diagnosis of CIN2 or three during colposcopy at DHMC [15]. To our knowledge, our study is the only study evaluating the follow-up for patients undergoing only cryotherapy for high-grade cervical dysplasia.

Our study's strengths include a standardized follow-up process in a closed health care system. DHMC also treats a diverse, lower-resource population with higher barriers to care. Limitations include the possibility that some patients obtained follow-up care outside our system. However, this is unlikely as the patients that DHMC serves typically do not access care elsewhere, as there are few other healthcare access points for this population within the Denver community. This suggests that most of these patients did not adhere to follow-up for this condition. Many sites across the United States no longer provide cryotherapy as a treatment option for CIN2+, potentially reducing this study's generalizability.

## Conclusions

In the most recent ASCCP guidelines, cryotherapy remains an acceptable option for treating high-grade cervical dysplasia. DHMC has utilized cryotherapy extensively for this indication as a less invasive option for patients who desire future childbearing. However, this study has prompted us to rethink the use of cryotherapy. Adherence to follow-up recommendations for the following cryotherapy for high-grade dysplasia within our system was poor. This is despite an established, standardized case management system for follow-up. Demographic factors were generally not associated with adherence to follow-up; therefore, no patient subgroup could be identified prior to the procedure that would suggest better adherence to follow-up. Given these findings, we will be utilizing cryotherapy only in unique situations.
